# Isolation and characterization of β-transducin repeat-containing protein ligands screened using a high-throughput screening system

**DOI:** 10.32604/or.2023.030240

**Published:** 2023-07-21

**Authors:** XINTONG LIU, EMIKO SANADA, JIANG LI, XIAOMENG LI, HIROYUKI OSADA, NOBUMOTO WATANABE

**Affiliations:** 1 Chemical Resource Development Research Unit, RIKEN CSRS, Wako, Saitama, 351-0198, Japan; 2 Department of RIKEN Molecular and Chemical Somatology, Graduate School of Medical and Dental Sciences, Tokyo Medical and Dental University, Bunkyo-ku, Tokyo, 113-8510, Japan; 3 Bioprobe Application Research Unit, RIKEN CSRS, Wako, Saitama, 351-0198, Japan; 4 Chemical Biology Research Group, RIKEN CSRS, Wako, Saitama, 351-0198, Japan; 5 Guangdong Engineering Research Center of Oral Restoration and Reconstruction, Affiliated Stomatology Hospital of Guangzhou Medical University, Guangzhou, 510180, China; 6 KingMed School of Laboratory Medicine, Guangzhou Medical University, Guangzhou, 510182, China; 7 Department of Pharmaceutical Sciences, University of Shizuoka, Suruga-ku, Shizuoka, 422-8526, Japan

**Keywords:** Ubiquitin, F-box protein, Chemical biology

## Abstract

β-transducin repeat-containing protein (β-TrCP) is an F-box protein subunit of the E3 Skp1-Cullin-F box (SCF) type ubiquitin-ligase complex, and provides the substrate specificity for the ligase. To find potent ligands of β-TrCP useful for the proteolysis targeting chimera (PROTAC) system using β-TrCP in the future, we developed a high-throughput screening system for small molecule β-TrCP ligands. We screened the chemical library utilizing the system and obtained several hit compounds. The effects of the hit compounds on *in vitro* ubiquitination activity of SCF^β-TrCP1^ and on downstream signaling pathways were examined. Hit compounds NPD5943, NPL62020-01, and NPL42040-01 inhibited the TNFα-induced degradation of IκBα and its phosphorylated form. Hence, they inhibited the activation of the transcription activity of NF-κB, indicating the effective inhibition of β-TrCP by the hit compounds in cells. Next, we performed an *in silico* analysis of the hit compounds to determine the important moieties of the hit compounds. Carboxyl groups of NPL62020-01 and NPL42040-01 and hydroxyl groups of NPD5943 created hydrogen bonds with β-TrCP similar to those created by intrinsic target phosphopeptides of β-TrCP. Our findings enhance our knowledge of useful small molecule ligands of β-TrCP and the importance of residues that can be ligands of β-TrCP.

## Introduction

Ubiquitination of substrates occurs between ubiquitin and the target protein [1]. This type of modification affects many cellular processes, including growth factor-induced endocytosis, DNA repair, and chromatin modification/transcriptional regulation [2]. E3 ligases are essential in the selection of substrates. Ubiquitin modification induces multiple types of cellular signaling; therefore, proper substrate selection ensures correct ubiquitin signaling [3]. The Skp1-Cullin-F box complex (SCF) is one of the classical types of E3 enzymes whose substrate specificity is determined by F-box proteins. As one of the F-box proteins, β-transducin repeat-containing protein (β-TrCP) is encoded by two different genes (FBXW1 and FBXW11), with indistinguishable biochemical identities in mammals [4,5]. β-TrCP, a substrate acceptor, along with CUL1, RING protein RBX1, and SKP1, forms the activated SCF^β-TrCP^ E3 ligase, essential for regulating protein levels of many key targets [6]. It recognizes the consensus degradation motif DSGφXS disruption motif (φ and X represent hydrophobic and arbitrary amino acids, respectively). The binding of β-TrCP depends on the phosphorylation of the two serines of the disruption motif, which links phosphorylated signaling to ubiquitination and destruction of proteins [7].

Examining the IκBα ubiquitination using TR-FRET, GS143 was identified as an inhibitor of β-TrCP, which suppressed β-TrCP dependent IκBα ubiquitination. GS143 inhibited the TNF-α-induced IκBα degradation and the NF-κB downstream reactions, excluding p53 and β-catenin responses. It is a potent inhibitor of the NF-κB signaling pathway [8]. In this study, GS143 was used as a positive control.

Proteolysis targeting chimera (PROTAC) is an engineered protein degradation technique [9]. PROTAC complex has been extensively studied globally in the field of cancer biology [10] and immunity [11], virology [12], and neurodegenerative diseases [13]. As protein degradation agents are used in clinical trials, the use PROTACS as a target protein-degradation system may prove to be useful. Bifunctional PROTAC molecules consist of a ligand for a protein of interest (POI) and a linker for an E3 ubiquitin ligase. Once bound to POIs, PROTACs can recruit E3 to ubiquitination and then proteasome degradation [14]. There are only 14 E3 ligases identified as the targets of PROTAC [15–28]; finding more target-binding ligands and E3 ligases suitable for PROTACs is essential.

In this study, we established a high-throughput screening system to screen β-TrCP ligands. Hit compounds were analyzed for their SCF^β-TrCP^ inhibitory activities. The hit compounds may be used for PROTAC construction in the future.

## Materials and Methods

### Cell culture

HEK293T and HeLa cells were cultured in Dulbecco’s Modified Eagle’s Medium (DMEM) (Gibco, Grand Island, NYS, USA), 10% Fetal Bovine Serum (FBS) (Biomeda, Saint Louis, MO, USA), and 1% penicillin/streptomycin (Gibco) in a humidified incubator at 37°C with 5% carbon dioxide. Express SF+ cells (Protein science, San Francisco, CA, USA) were cultured in serum-free medium complete (Gibco) and penicillin/streptomycin (Gibco) in an incubator at 28°C on the shaker.

### High-throughput screening system for β-TrCP ligands

*Not*I digested mAG-β-TrCP1 (mAG, N-terminally fused fluorescent monomeric Azami Green [29]) fragment was inserted to *Not*I pFASTBacI (Gibco) site for obtaining recombinant plasmid containing mAG-β-TrCP1. The recombinant plasmids were then transformed into DH10Bac cells and the obtained recombinant bacmids were transfected into Express SF+ Cells. Phosphopeptides C-IkB pp15 with target sequences (seq: CDDRHDpSGLDpSMKDEE) for β-TrCP-dependent binding or its negative control C-IkB s15 (seq: CDDRHDSGLDSMKDEE) were used in the establishment of the high-throughput system. These phosphopeptides, and their non-phosphorylated derivatives, were chemically synthesized at RIKEN CBS, Research Resources Center, Japan. C-IkB pp15 or C-IkB s15 (100 μL/well of 10 μM solution) were covalently attached to maleimide-activated 96-well plates (Themo Fisher Scientific, Waltham, MA, USA) overnight. Furthermore, the plates were washed using PBS, and unreacted maleimide was blocked using 0.01 mg/ml L-cysteine at 4°C for over 1 h. mAG-β-TrCP1 was expressed in Express SF+ cells. The cells were lysed by sonication in sonication buffer (20 mM Tris pH 7.5, 125 mM NaCl, 0.5% Nonidet P-40, 1 mM EDTA, 1 mM dithiothreitol, 200 μM Na_3_VO_4_, 50 mM NaF) supplemented with complete protease inhibitors (Roche life Science, Mannheim, BW, GER). After centrifugation (4°C, 9,100 × *g*, 10 min), and the lysate with mAG-β-TrCP1 protein was mixed with GS143 (Sigma Aldrich, USA) and other test compounds (RIKEN NPDepo chemical library). All the compounds were examined at 0.2 mg/mL in the first screening, and 0.2 mM for further confirmation. The mixture was placed in wells of the plate and incubated at 4°C overnight. After washing, bound mAG-β-TrCP1 was quantitated using spectrofluorometry (excitation, 470 nm; emission, 525 nm).

### In vitro ubiquitination assay

Components of SCF complex (SKP1, Cul1, Rbx1, and hemagglutinin (HA)-tagged β-TrCP) were expressed in HEK293T cells. Anti-HA antibody was added into the SCF complexes expressed HEK 293T cell lysate and rotated at 4°C for 1 h. Aliquots of this mixture were added to protein G beads and rotated at 4°C for 30 min. After washing the beads in cell lysis buffer three times, the tube was centrifuged at 20,400 × *g* for 5 min and the supernatant was discarded. The protein on the beads was used as recombinant SCF^β-TrCP^ complexes. Aliquots of SCF complexes bound on the beads were then added to ubiquitination reactions (10 mM Tris pH = 7.5, 5 mM MgCl_2_, 1 mM DTT, 1 mM ATP, 20 mM creatine phosphate, 0.1 mg/mL creatine kinase, 5 mM NaF, 1 mM Na_3_VO_4_, 2 mg/mL ubiquitin, 0.5 μg E1 enzyme, 2 μg E2 enzyme) in a final volume of 30 µL. Compounds of 100 µM final concentration were added to reactions, and GST-Wee1KR was added as substrate 15 min later. The reaction mixtures were incubated at 37°C for 2 h and were further examined by immunoblotting.

### Immunoblotting

Immunoblotting followed by *in vitro* ubiquitination assay was performed using anti-human Wee1A [30]. Immunoblotting in the analysis of protein degradation was performed using anti-phospho-β-catenin (Ser33/37/Thr41) antibody (1:1000, Cell Signaling Technology, Danvers, MA, USA), anti-phospho-IκBα (Ser32) (14D4), Rabbit mAb (1:1000, Cell Signaling Technology), anti-β-catenin (D10A8) (1:1000, Cell Signaling Technology), anti-IκBα antibody (1:1000, Cell Signaling Technology), and Goat polyclonal anti-rabbit IgG antibody (1:5000, Proteintech, Am Klopferspitz, Planegg-Martinsried, Germany). The transferred membrane was incubated with specific antibody, then detected using Super Signal West Pico chemiluminescent substrate (Thermo Fisher Scientific) and FUSION SOLO S (VILBER LOURMAT/MS equipment, Zi Sud torcy, Marne-la-vallee, France). The quantification of immunoblotting results was performed using ImageJ software.

### Analysis of protein degradation

For examining the effect on degradation of IκBα, HeLa cells were cultured in the presence or absence of the compound for 30 min. TNF-α (Cat. No. TNA-H4211, Acro Biosystems, Newark, DE, USA) was added (40 ng/mL) and incubated for another 5 min. The cell extracts were analyzed using immunoblotting using antibodies against IκBα and phosphorylated IκBα (p-IκBα).

For examining the effect on degradation of β-catenin, HeLa cells were cultured in the presence or absence of the compound for 3 h. The cell extracts were examined by immunoblotting using antibodies against β-catenin and phosphorylated β-catenin (p-β-catenin).

### Luciferase reporter assays

HeLa cells were seeded on 96-well plates. Subsequently, TCF and NF-κB transcription activity was examined by the luciferase assay system. T-cell Factor (TCF) transcription activity was assessed by TOP/FOP-Flash luciferase reporter assay, using a pRL-SV40 vector as an internal control. TOP FLASH contains three TCF transcription factors and a firefly luciferase reporter with a minimal promoter and an upstream of the luciferase reporter gene. FOP FLASH is similar to TOP reporter; however, it has three mutated TCF-binding sites and is used as a negative control. The average ratio of TOP to renilla luciferase divided by FOP was used as TCF activity. TOP flash (Sigma-Aldrich, Temecula, CA, USA) or FOP FLASH reporter plasmids (Sigma-Aldrich) and pRL-sv40 renilla luciferase reporter (Promega) were co-transfected into HeLa cells using Lipofectamine 3000 reagent kit (Thermo Fisher Scientific). Furthermore, 24 h after transfection, cells were cultured with the compounds of different dilutions for another 24 h. The cells were lysed, and the luminescence was measured using the Luciferase Assay System (Promega). A minimum of three samples were taken for each sample.

For NF-κB transcription activity, PGL-4.32 contains five copies of the NF-κB response element, which is responsible for the transcription of the luciferase reporter gene, with pRL-SV40 as the internal control. NF-κB transcription activity was determined by firefly luciferase expression and normalized by renilla luciferase level. pGL-4.32 reporter plasmids (Promega) and pRL-sv40 renilla luciferase reporter (Promega) were co-transfected into HeLa cells using Lipofectamine 3000 reagent kit. Subsequently, 24 h after transfection, cells were cultured with the compounds of different dilutions for another 24 h. Furthermore, TNF-α was added, and the plates were cultured for another 4 h. The cells were lysed, and the luminescence was measured using the Luciferase Assay System. A minimum of three samples were taken for each sample.

### Molecular docking simulation

The 3D structures of the compounds were obtained from the NCBI PubChem database (https://pubchem.ncbi.nlm.nih.gov/). The SDF file is transformed into PDB by PyMol software, and the Autodock tools-1.5.7 tool converts it into PDBQT format. The crystallographic data of β-TrCP1 (PDB code 1P22, PDB format) were obtained from Protein Data Bank and Chimera 1.16 software. The PDBQT files were created using the Autodock tool 1.5.7. Molecular docking simulation proceeded by executing the autodock vina algorithm. A total of 150 flexible docking runs, an energy range of 5, and 20 number modes were set, and the affinities (Kcal/mol) were calculated. Consequently, all the figures were made using PyMol software.

### Statistical analysis

Luciferase reporter assay was examined in triplicate. The data were presented as the average ± S.D. GraphPad Prism 9.4.1 software (Macintosh Version by Software MacKiev 1994–2022 GraphPad Software, LLC) was used to evaluate the statistical differences among the groups by one-way analysis of variance (ANOVA).

## Results

### Establishment of the high-throughput system and first screening for β-TrCP ligands

**Figure 1:**
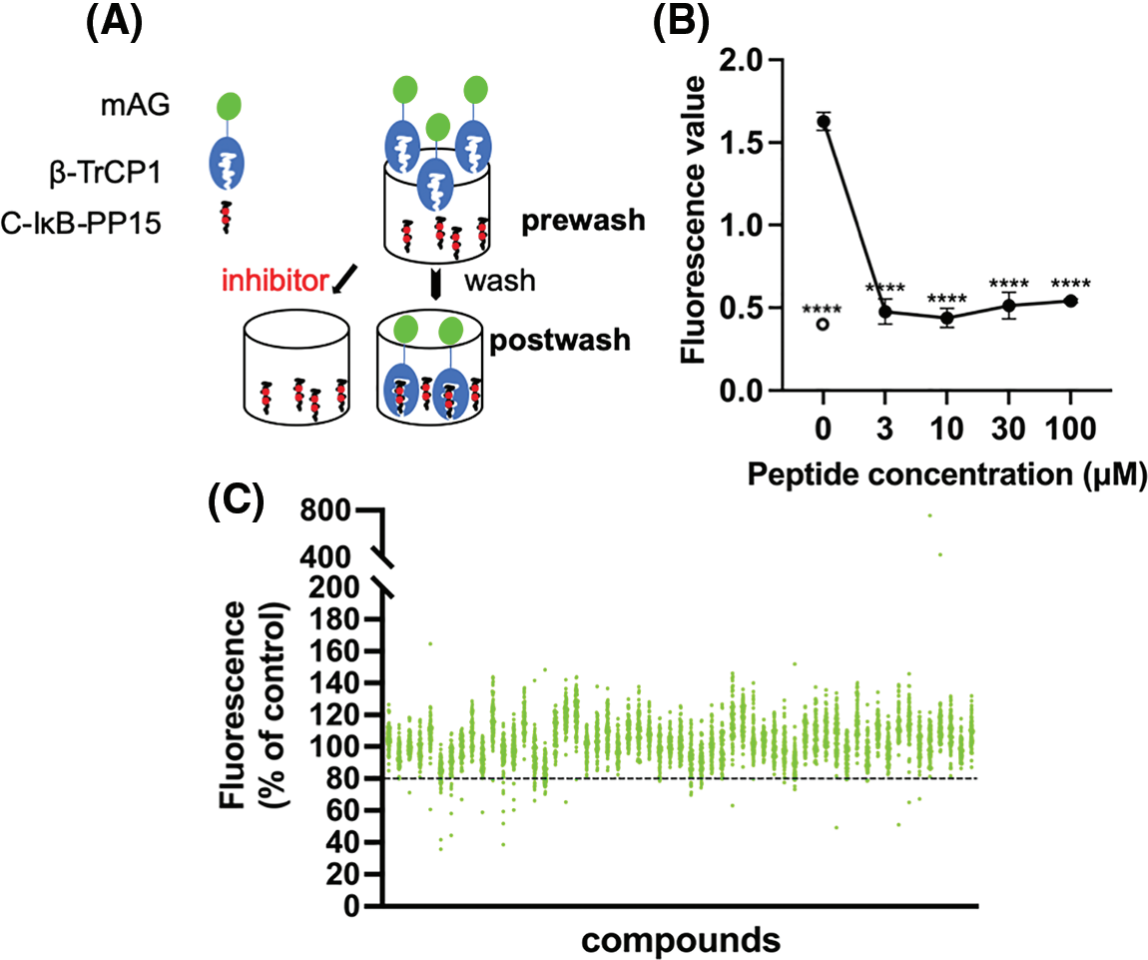
Screening system and high-throughput screening for ligands of β-TrCP. (A) Insect cell lysates expressing mAG-β-TrCP1 were added to 96-well plates that are covalently attached with IκB phosphopeptides. In the presence of the small molecules competing with the phosphopeptides for mAG-β-TrCP binding, the fluorescence of the plate decreases. (B) The phosphopeptide of β-TrCP binding sequence (C-IκB-PP15) and unphosphorylated version (C-IκB-s15) were bound to 96-well plates. The binding of mAG-β-TrCP1 to C-IκB-PP15 (closed circle) and C-IκB-s15 (open circle) was determined using spectrofluorometry. A concentration-dependent binding decrease was observed in the presence of the free IκB phosphopeptides (C-IκB-PP15). (The data are presented as the average ± S.D. The statistical differences among the groups were evaluated using one-way ANOVA. *p*-value: **** < 0.0001) (C) Results of initial screening of ~5,000 compounds. The effect of each compound (at 0.2 mg/mL) on mAG-β-TrCP1 binding was standardized and is represented as a percentage value of control (100% indicating no inhibition). The compounds that inhibited the binding by >20% were the initial hits.

C-IκB-PP15 is a phosphopeptide whose sequence is derived from IκB that is known to bind to β-TrCP. C-IκB-s15 is its non-phosphorylated version, which cannot bind to β-TrCP and was used as a negative control. They were both bound to the maleimide-activated plate using N-terminal cysteine. Express SF+ cell lysates expressing mAG-β-TrCP1 were added into the 96-well plate. After enough binding and washing, the bound mAG-β-TrCP1 was quantitated using spectrofluorometry. The postwash value indicates the binding of mAG-β-TrCP1 and C-IκB-PP15. When the small molecules compete with the phosphopeptides for mAG-β-TrCP1 binding, there was less plate fluorescence (Fig. 1A).

The free target phosphopeptide competitively inhibited the binding of the mAG-β-TrCP1 to the plate-fixed phosphopeptide. The efficacy of the system was confirmed by mixing serially diluted phosphopeptide into the cell lysate (Fig. 1B). To identify potent ligands of β-TrCP, ~5,000 RIKEN Natural Products Depository (NPDepo) compounds were examined using this high-throughput screening system in a final concentration of 0.2 mg/mL and 109 small molecules were identified as initial positive compounds inhibiting binding by >~20% (Fig. 1C). Then, 109 compounds were re-screened at a lower concentration (0.2 mM), and 13 hits were obtained. Furthermore, 372 derivatives of these 13 initial hits and 27 derivatives of known inhibitor GS143 were examined. Finally, 23 positive NPDepo compounds (13 hits + 10 positive derivatives) and 13 positive GS143 derivatives were identified from the high-throughput system.

### The secondary screening-in vitro ubiquitination assay

As the secondary screening, the activity of hit compounds was examined using the SCF^β-TrCP1^ ubiquitination activity *in vitro* at a final concentration of 100 µM. The kinase-negative mutant GST-Wee1 KR was used as the substrate of SCF^β-TrCP1^.

**Figure 2:**
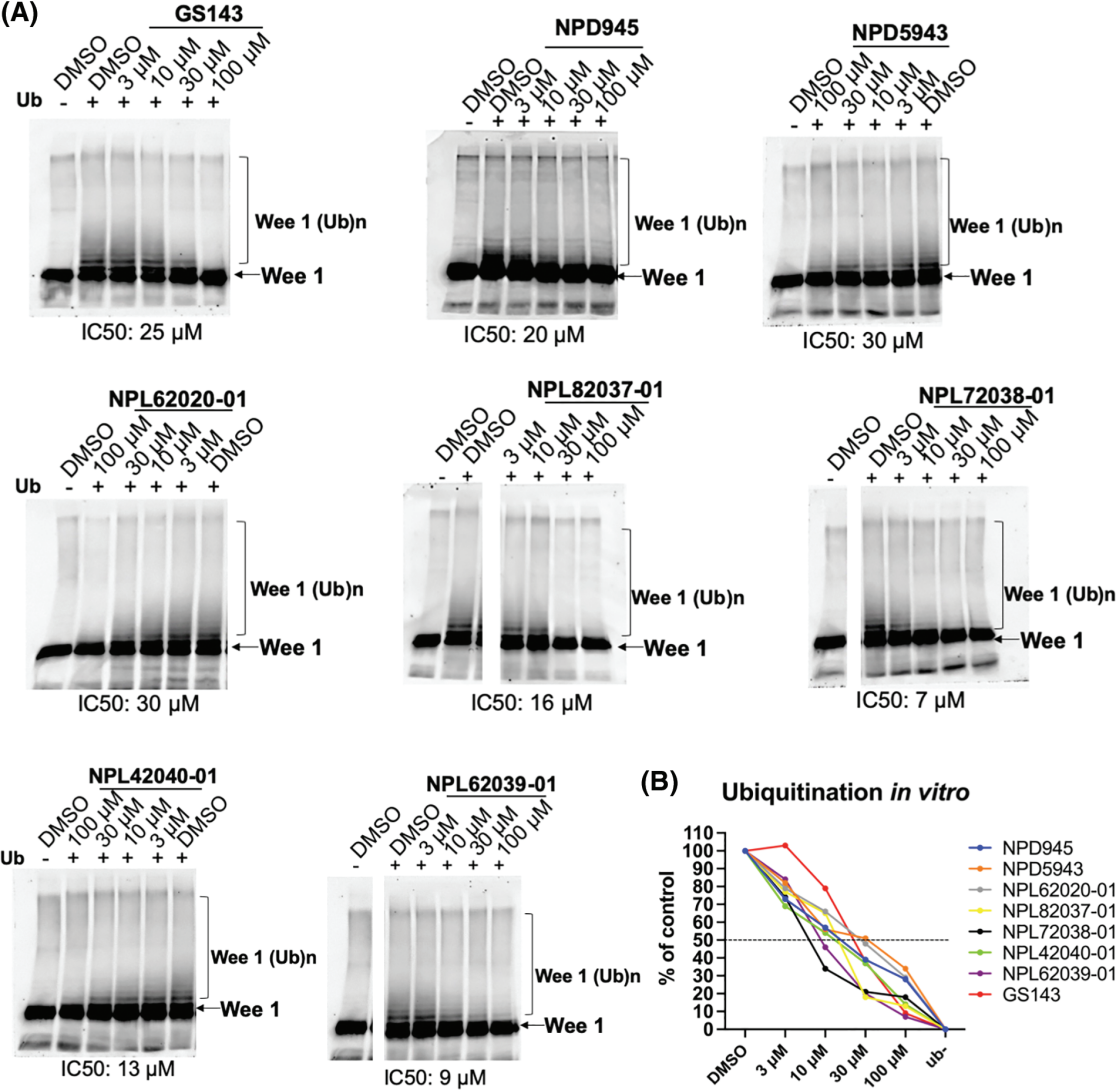
Dose-dependent inhibition of ubiquitination by hit compounds of Wee1KR. In the presence of these seven compounds, *in vitro* ubiquitination of Wee1KR is inhibited in a dose-dependent manner (IC 50 of each compound obtained in (B) is shown) (B) The results in (A) were quantitated by ImageJ and plotted.

The results indicate that eight compounds from NPDepo hits and 10 GS143 derivatives decreased the ubiquitination of Wee1 by competitive binding with β-TrCP1. The dose-dependent inhibitory activity of these 18 compounds (8 NPDepo compounds + 10 GS143 derivatives) was further examined using *in vitro* ubiquitination system. Two NPDepo hit compounds (NPD5943 and NPD945) and five positive GS143 derivatives (NPL72038-01, NPL82037-01, NPL62020-01, NPL42040-01, and NPL62039-01) were selected as the most potent (Fig. 2A). The IC50 value of GS143, GS143 derivatives and NPDepo hit compounds are GS143 (IC50 = 25 µM), NPL42040-01 (IC50 = 13 µM), NPL62039-01 (IC50 = 9 µM), NPL72038-01 (IC50 = 7 µM) and NPL82037-01 (IC50 = 16 µM), NPD5943 (IC50 = 30 µM), NPD945 (IC50 = 20 µM), and NPL62020-01 (IC50 = 30 µM) (Fig. 2B).

### Effect of the hit compounds on Wnt/β-catenin signaling pathway

**Figure 3:**
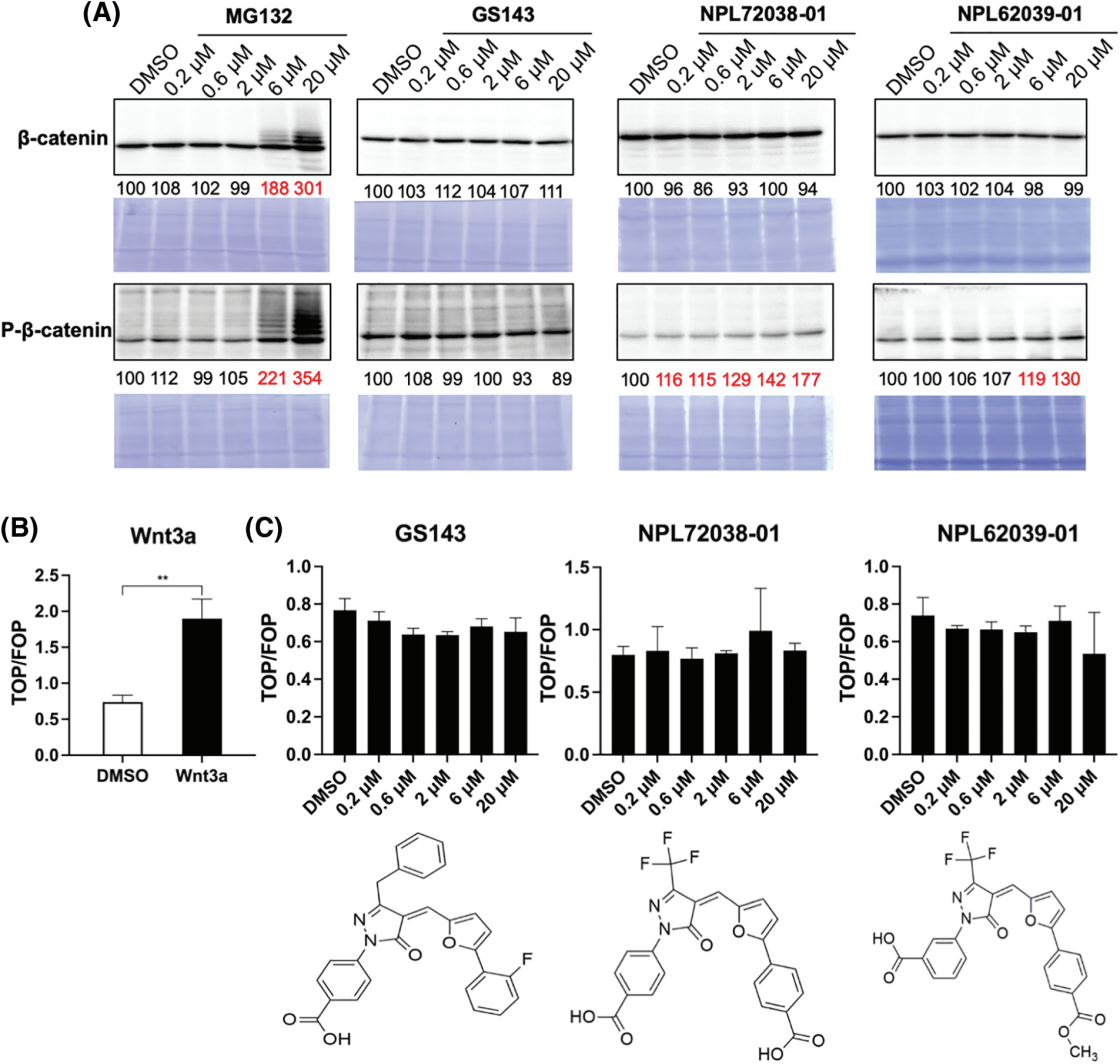
NPL72038-01 and NPL62039-01 accumulated phosphorylated β-catenin; however, they did not significantly affect the β-catenin signaling pathway. (A) Effect of MG132, GS143, and two positive compounds on the levels of β-catenin and phosphorylated β-catenin in HeLa cells. The levels are quantitated and shown under the panel as % of DMSO control. Increased values (>115) are shown in red. (B) Wnt3a (100 ng/mL) increased the level of TCF transcription. (C) Effect of GS143 and two compounds on TCF transcription in HeLa cells. The data are presented as the average (n = 3) ± S.D. The statistical differences among the groups were evaluated using one-way ANOVA. (*p*-value: ** < 0.01).

In the classic Wnt signaling pathway, when β-TrCP induced ubiquitination is inhibited, β-catenin accumulates and further activates nucleolar TCF transcription [31,32]. We examined the effects of hit compounds on β-catenin degradation in HeLa cells. HeLa cells were cultured for 3 h with or without the compound. The levels of β-catenin and p-β-catenin were examined in the cell extracts. Proteasome inhibitor MG132 caused the accumulation of β-catenin and phosphorylated form, while the GS143 did not affect β-catenin and p-β-catenin, which was similar to the published results [8]. Among the seven hit compounds identified from the screening above, only GS143 derivatives NPL72038-01 and NPL62039-01 promoted the accumulation of phosphorylated β-catenin. However, they did not significantly affect the β-catenin level (Fig. 3A).

Wnt/β-catenin signaling pathway activity was also assessed using TOP/FOP-Flash luciferase reporter assay. The average ratio of TOP to renilla luciferase divided by FOP was used as TCF activity. Wnt3a is the molecule that stimulates classic Wnt signaling [11]. After adding Wnt3a, the level of TCF transcription increased, which confirmed the efficacy of this system (Fig. 3B). Similarly, in the immunoblotting result obtained, GS143 did not affect TCF transcription and was inhibited minimally. NPL72038-01 and NPL62039-01 also did not cause a high increase in TCF transcription (Fig. 3C).

### Effect of the hit compounds on the NF-kB signaling pathway

**Figure 4:**
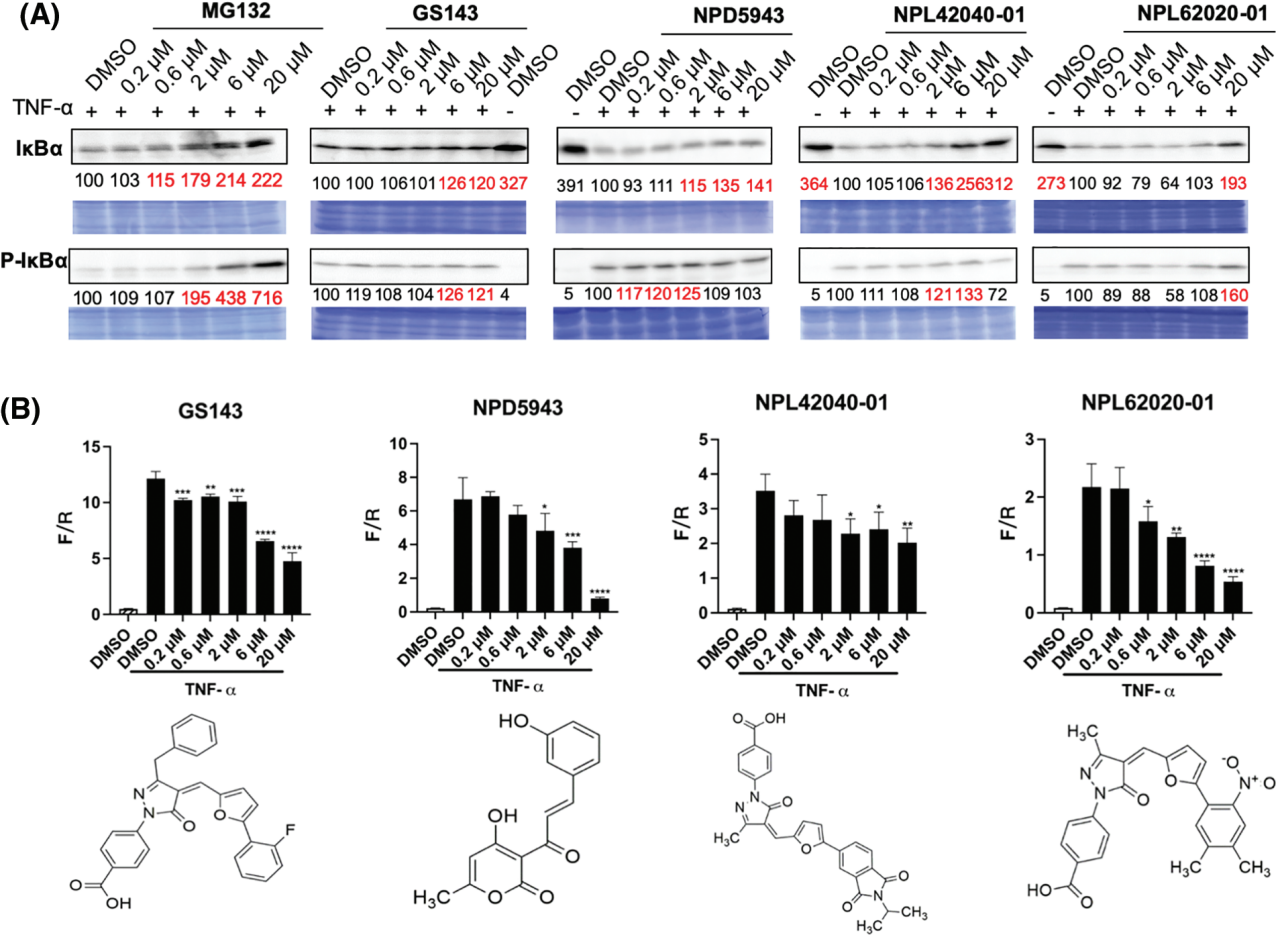
NPD5943, NPL42040-01, and NPL 62020-01 show an inhibitory effect in the NF-κB signaling pathway in a dose-dependent manner. (A) Effect of MG132, GS143, and three compounds on TNF-α induced IκBα degradation in HeLa cells. The levels of IκBα and phosphorylated IκBα in HeLa cells after the treatment are shown. The levels are quantitated and shown under the panel as % of DMSO control. (B) Effect of three positive compounds on TNF α induced NF-κB activation of transcription in HeLa cells: Samples were examined in triplicate. The data are presented as the average ± S.D. The statistical differences among the groups were evaluated using one-way ANOVA (*p*-value: **** < 0.0001, *** < 0.001, ** < 0.01, * < 0.05).

NF-κB is considered a typical pro-inflammatory signaling pathway, mediated by the effect of pro-inflammatory cytokines like TNF-α. TNF-α induces the β-TrCP-dependent ubiquitination and degradation of IκBα and relocates NF-κB into the nucleus [33,34]. The impacts of hit compounds on IκBα degradation were further examined in HeLa cells. Cells were cultured for 30 min with or without the indicated compounds. Then, TNF-α was added and incubated for an extra 5 min. The level of IκBα and p-IκBα was examined in cell extracts. MG132 and GS143 caused the inhibition of the degradation of IκBα and its phosphorylated form, just as published [34]. Among the seven hit compounds from secondary screening, NPDepo compound NPD5943 and GS143 derivatives NPL72038-01, NPL62020-01, and NPL42040-01 increased the level of IκBα and p-IκBα (Fig. 4A).

NF-κB transcription activity was also determined by firefly luciferase expression normalized by renilla luciferase level. TNF-α activated the transcription of NF-κB. Among the seven potent compounds from the secondary screening, the treatment of NPD5943, NPL62020-01, and NPL42040-01 reduced NF-κB activation dose-dependently. NPL82037-01 and NPD945 also caused accumulation of TNF-α induced IκBα and p-IκBα level. However, NPL82037-01 caused a high cell death at high concentrations, and NPD945 did not affect NF-κB transcription activity. These two compounds were not regarded as effective in NF-kB signaling pathway. GS143 at 20 μM inhibited the transcription to ~40% of that of the control group (DMSO, TNF-α). NPD5943 and NPL62020-01 can inhibit it to approximately 20% of that of the control at 20 μM, which shows better inhibition than GS143 at high concentration (Fig. 4B).

The compounds that exhibit inhibitory effect on either the β-catenin or NF-κB pathways illustrate their binding ability to β-TrCP E3 ligase, and can be considered as hit ligands. Therefore, although these compounds (NPD5943, NPL42040-01, and NPL62020-01) do not have noticeable effects on the β-catenin pathway, we still regarded them as the positive ligands because of their significant effects on the NF-kB pathway. However, NPL72038-01 and NPL62039-01 did not exhibit significant effect in either β-catenin or NF-kB signaling pathway; hence, we did not regard them as hit compounds.

### In silico analysis of the hit compounds

The structures of the three hit compounds were further analyzed using *in silico* analysis to examine the important moieties and explain their activity. Molecular docking and binding site analysis of β-TrCP (PDB:1P22) were carried out with hit compounds using the docking program autodock vina and PyMOL software. The results were achieved by extensively using autodock tools for setting up docking runs. The meta information, including the docking score, is displayed for each pose in an automatically recorded text file. The sorted list of docking ligands and their respective combined poses can be exported.

**Figure 5:**
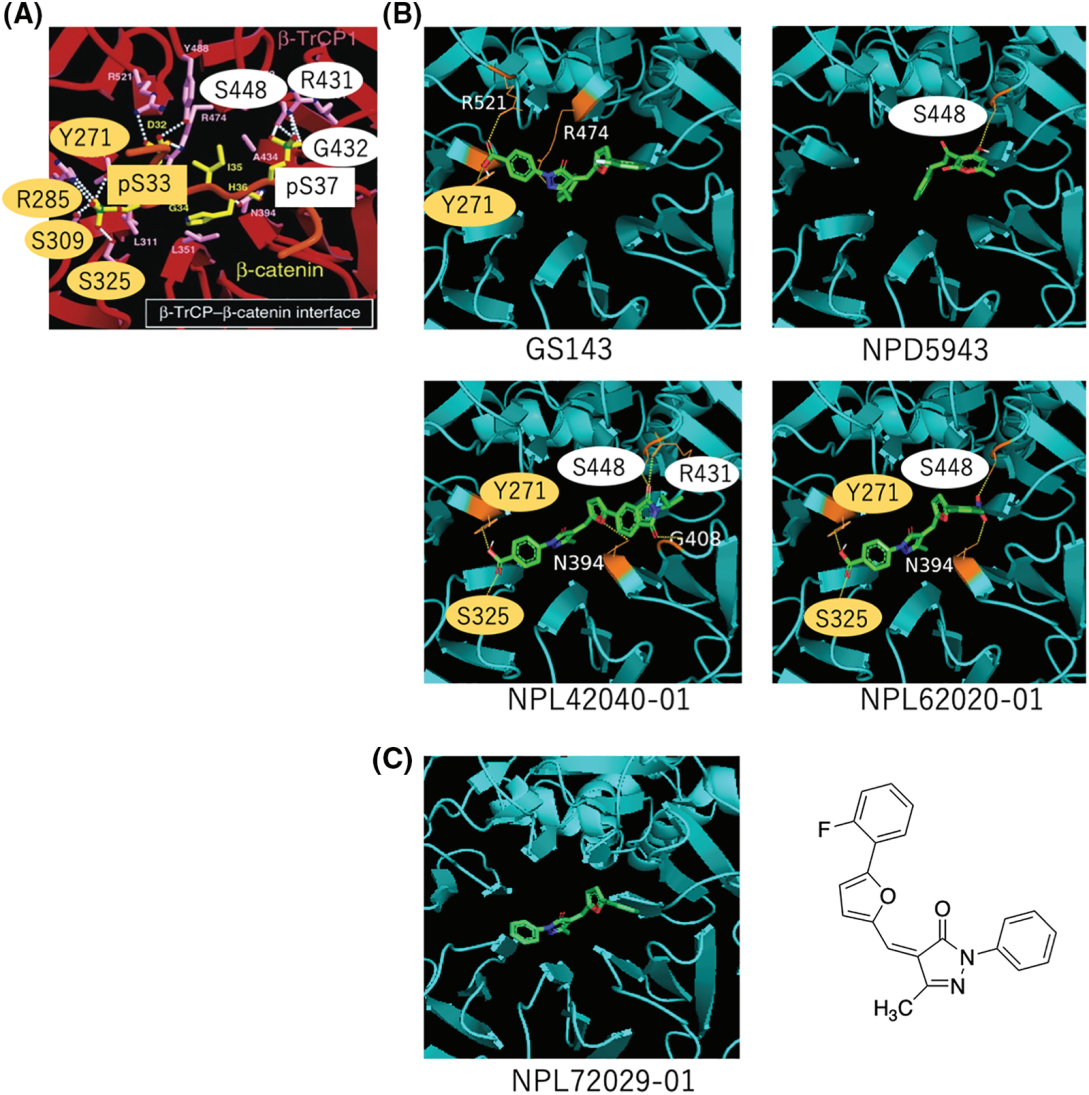
*In silico* analysis of the hit compounds. (A) The interface of phosphorylated β-catenin (pS33, pS37) and β-TrCP: β-TrCP is shown in red, with its side chains in pink, and the phosphodegron peptides of β-catenin are in yellow (phosphate groups on serines are shown in green and red). White dotted lines represent H bonds. Amino acid residues linked to pS33 of β-catenin and those to pS37 are marked in yellow and white circles, respectively. These phosphoamino acids of β-catenin, pS33 and pS37 are marked in yellow and white boxes, respectively. The original figure from [7] was modified. (B) The interface of hit compounds and β-TrCP: β-TrCP is shown in light blue. When amino acids shown in (A) create an H bond with the compound, they are shown in the same color as in (A). (C) The interface of negative compound (NPL72029-01) and β-TrCP: β-TrCP is shown in light blue. The structure of NPL72029-01 is shown on the right.

Reportedly, β-TrCP recognizes a specific motif with phosphorylated serines [7]. Their interface indicated the hydrogen bond (H bond) binding to the phosphorylated site. The amino acids bound to pS33 (Y271, R285, S309, and S325) are marked in a yellow circle, and the amino acids bound to pS37 (S448, R431, and G432) are marked in a white circle (Fig. 5A). We focused on these amino acids for the H bond formation with the compounds.

The result shows GS143 created three H bonds with β-TrCP. One binding amino acid of the H bond created by carboxyl groups is similar to β-catenin pS33 binding amino acid (Y271), indicating the importance of the carboxyl groups. By comparing the structures of the three hit compounds, we noticed that the carboxyl groups of the two GS143 derivatives and the hydroxyl group of NPD5943 created H bonds when bound to β-TrCP. The two active GS143 derivatives created more H bonds than GS143, and they have the same binding sites with both pS33 and pS37 (NPL42040-01: Y271, S325, S448, R431; NPL62020-01: Y271, S325, S448). Among them, NPL42040-01 is the most effective in immunoblotting results; it created a total of six H bonds in the binding, and four of their binding residues belong to both pS33 and pS37 (Fig. 5B).

To further explore the importance of carboxyl groups, we analyzed another negative ligand (NPL72029-01) that does not have the carboxyl groups. The result shows that NPL72029-01 did not create any H bonds to β-TrCP, which indicates the importance of carboxyl to β-TrCP binding.

## Discussion

A significant level of SCF^β-TrCP^ is available in the cells, showing enormous potential to be a target of PROTAC [31]. β-TrCP can potentially be a competitive target of the PROTAC technique. A sensitive and rapid screening system may provide more possibilities in the synthesis.

In this study, we established a high-throughput screening system by constructing recombinant baculoviruses to express fluorescent fusion protein mAG-β-TrCP1 and identified the ligands of β-TrCP with it. The high-throughput screening revealed 36 compounds that show competitive binding activity to β-TrCP. *In vitro* ubiquitination assay re-examined these compounds; seven were obtained as hit compounds. The effect of the hit compounds on substrate β-catenin and IκBα degradation was further examined to reflect the competitive binding activity to β-TrCP.

Results revealed that a majority of the hit compounds caused marked intracellular accumulation of IκBα and its phosphorylated form, similar to the effect of the proteasome inhibitor MG132. Three of the hit compounds showed an inhibitory effect on TNF-α induced NF-κB transcription in a dose-dependent manner, which proved their effectiveness in the NF-κB signaling pathway. As GS143 is a specific inhibitor of β-TrCP [8], NPL62020-01 and NPL42040-01 were also predicted to be specific to β-TrCP. As for NPD5943, we examined its binding to another F-Box protein of SCF-type E3 ligase, SKP2. As expected, NPD5943 did not bind to SKP2 at all (data not shown). In addition, because β-TrCP binds to phosphorylated peptides, it is possible that its ligand can bind to other proteins that bind to phosphorylated proteins. The polo-box domain of Plk1, 14-3-3 proteins, and Pin1 are examples of such protein. When we examined the binding of NPD5943 to such proteins, we did not observe any binding (data not shown). These results suggest that the binding of NPD5943 to β-TrCP is highly specific. We also examined the non-specific cytotoxicity of NPD5943, NPL62020-01, and NPL42040-01. We found that these compounds showed cytotoxicity only at high concentration (data not shown). As inhibition of β-TrCP by siRNA did not show any cytotoxicic effects on HeLa cells, these results also support the fact that these hit compounds do not have any non-specific inhibition to other proteins.

A majority of the hit compounds that affect the degradation of IkBα did not result in the accumulation of β-catenin, the E3 of which is identical to the SCF^β-TrCP1^ of IκBα. Moreover, the two hit compounds that inhibited the p-β-catenin degradation did not increase β-catenin itself, indicating that only a small population of β-catenin was increased by these compounds. We speculate that this increase may not be sufficient to promote TCF transcription. Thus, we could not find compounds that inhibited the β-catenin degradation.

Corroborating with findings of a previous study, GS143 also showed similar behavior that explicitly targets IkBα degradation, excluding β-catenin [8]. GS143 was identified by examining the IκBα ubiquitination using TR-FRET. This effect of GS143 was more likely to be a physical interaction with p-IκBα and SCF^β-TrCP1^, the disruption of which inhibits IκBα ubiquitination [8]. However, in this study, these compounds were screened using binding and *in vitro* ubiquitination assays, which confirmed their competitive binding activity to β-TrCP. We hypothesized that such an effect may be because their chemical moieties result in different binding characters.

GS143 has been reported as a novel NF-κB signaling inhibitor with great potential in the treatment of air­way inflammation in asth­matic patients [34]. Here, we found more potent compounds that function better than GS143 in the inhibition of TNF-α induced IkBα degradation and the activation of NF-κB. The hit compounds obtained here showed potent effect in NF-κB signaling, and did not increase the level of β-catenin, which can be candidate compounds with anti-inflammatory and anti-tumor properties. This possibility can be explored in future studies.

*In silico* analysis summarized the important moieties and explained why the three hit compounds are active. According to the results, when GS143 binds to β-TrCP, the carboxyl moiety of GS143 has two hydrogen bonds; one had the same occupation as the amino acid of β-TrCP that was created with β-catenin pS33. The carboxyl moiety of the two GS143 derivatives also created hydrogen bonds when bound to β-TrCP, while the negative compound without carboxyl groups did not. These results may explain its importance in these GS143 derivatives. Hydroxyl groups of NPD5943 also play an important role in H bond creation. These residues should be protected in the synthesis of PROTAC. All the two active GS143 derivatives have binding residues that belong to pS33 and pS37. We presumed GS143 derivatives with more H bond occupations of pS33 and pS37 binding amino acids may show high activity in β-TrCP binding.

In summary, we developed a flexible, sensitive and, specific system that can be used to screen β-TrCP ligands. The potent ligands for β-TrCP were finally obtained as expected, which can be used as tools for PROTAC construction in the future. PROTAC technology has significant advantages over conventional drugs, and has shown great potential as a therapeutic and biological tool. This study provides a new prospect of the target E3 ligase selection and promising binding ligands of PROTACs construction. The potent ligands can be used as elements of the PROTAC complex that recruits β-TrCP to the protein of interest, which may prove great potentiality in clinic therapy in the future.

## Data Availability

The article includes all the data supporting the conclusions of this manuscript. The corresponding author can be contacted for raw data requests.
